# Caring Beyond the Challenges: Grit in Dementia Family Caregivers

**DOI:** 10.1093/geroni/igab046.030

**Published:** 2021-12-17

**Authors:** Rosemary Blieszner, Jyoti Savla, Karen Roberto, Brandy McCann, Emily Hoyt

**Affiliations:** Virginia Tech, Blacksburg, Virginia, United States

## Abstract

Scholars and practitioners recognize the importance of family caregivers for persons with dementia (PwD) persevering through difficulties and remaining committed to providing care (i.e., possessing grit). Based on Pearlin’s stress process model, we examined how grit is associated with stressors and strains that interfere with caregiver well-being and jeopardize continued caregiving. The sample included 158 family caregivers of PwD from rural Appalachia. SEM analysis revealed that grit and family and friend affectual solidarity contributed significantly to mastery. Grit and family solidarity were associated indirectly with role overload through their effect on mastery. Results demonstrate the value of acknowledging the role of grit in enhancing caregivers’ confidence about managing difficulties they face and reducing their sense of being overwhelmed by caregiving responsibilities. Thus, strengthening dementia caregivers’ commitment to and perseverance in their role is crucial for sustaining their motivation to provide care, despite the challenges they face.

